# Histological verification of the treatment effect of tirabrutinib for relapsed/refractory primary central nervous system lymphoma

**DOI:** 10.1186/s40164-021-00222-5

**Published:** 2021-04-26

**Authors:** Yoshiko Okita, Rieko Kano-Fujiwara, Shin-Ichi Nakatsuka, Keiichiro Honma, Manabu Kinoshita

**Affiliations:** 1 Department of Neurosurgery, Osaka International Cancer Institute, 3-1-69 Otemae, Chuo-ku, Osaka, 541-8567 Japan; 2 Department of Diagnostic Pathology and Cytology, Osaka International Cancer Institute, 3-1-69 Otemae, Chuo-ku, Osaka, 541-8567 Japan; 3 Present Address: Department of Neurosurgery, Asahikawa Medical University, Midorigaoka-higashi 2-1-1-1, Asahikawa, Hokkaido 078-8510 Japan

**Keywords:** Primary central nervous system lymphoma, Tirabrutinib, Bruton’s tyrosine kinase inhibitor, Autopsy

## Abstract

Tirabrutinib (ONO/GS-4059; Ono Pharmaceutical) is a newly developed drug that selectively and irreversibly inhibits Bruton’s tyrosine kinase (BTK) and has been approved in Japan for treating relapsed/refractory primary central nervous system lymphoma (PCNSL). However, its therapeutic effect is yet to be verified at the pathological level in human patients. A 64-year-old patient with recurrent PCNSL enrolled in the phase I/II clinical trial of tirabrutinib, a second-generation BTK inhibitor designed for treating relapsed/refractory PCNSL. The left cerebellum lesions on magnetic resonance imaging disappeared one month after tirabrutinib treatment. The patient died because of suspected pneumocystis pneumonia and acute exacerbation of interstitial pneumonia 43 days after starting tirabrutinib. An autopsy confirmed no viable tumor cells in the entire brain, including the left cerebellum lesion, confirming complete obliteration of tumor cells by tirabrutinib. This letter pathologically confirms the effect of tirabrutinib on relapsed/refractory PCNSL for the first time in humans.

*Trial registration*: JapicCTI-173646. Registered 14 July 2017, https://www.clinicaltrials.jp/cti-user/trial/ShowDirect.jsp?japicId=JapicCTI-173646.

To the Editor,

Primary central nervous system lymphoma (PCNSL) is a highly aggressive non-Hodgkin’s lymphoma associated with a poor prognosis. The optimal treatment for patients with recurrent PCNSL has not yet been established. Tirabrutinib (ONO/GS-4059; Ono Pharmaceutical) is a second-generation, potent, highly selective Bruton’s tyrosine kinase (BTK) inhibitor with less off-target effect compared to those of the first-generation [1] Although it was approved in Japan for treating relapsed/refractory PCNSL [2], its therapeutic effect is yet to be verified at the pathological level in human patients. We herein report a patient with recurrent PCNSL treated by tirabrutinib from whom we obtained post-treatment tissues after autopsy. This report is the first to describe the pathology of recurrent PCNSL following tirabrutinib treatment.

A 64-year-old woman with a diffuse large B cell lymphoma (CD20(+), CD79α(+), CD3(−), CD5(+), CD30(−)) (Fig. 1a) in the right frontal lobe was initially treated with three high-dose methotrexate chemotherapy and radiotherapy (36 Gy with a local boost of 9 Gy) followed by multiple stereotactic radiotherapies at tumor recurrence. A fourth recurrence occurred in the left occipital lobe three years after the biopsy. Although stereotactic radiotherapy was again prescribed, the patient further experienced impaired consciousness, and magnetic resonance imaging (MRI) revealed a disseminated contrast-enhancing lesion in the left cerebellum. The patient enrolled in the phase I/II clinical trial of tirabrutinib at this point [2].

**Fig. 1:**
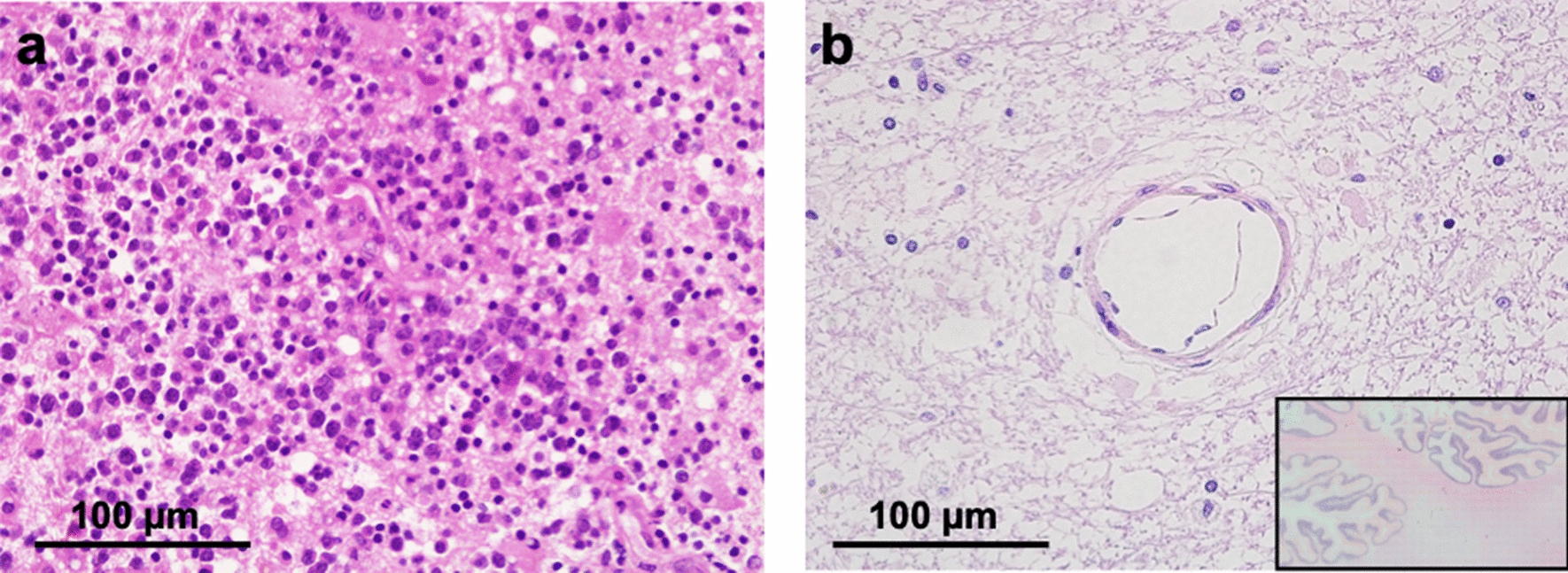
Histological findings of brain biopsied samples of the initial lesion and that after tirabrutinib treatment are shown. The initial lesion was diagnosed as diffuse large B cell lymphoma (DLBCL), as shown in hematoxylin and eosin stain (**a**). Histological appearance of an autopsied specimen of the lesion after tirabrutinib treatment showed no evidence of viable cells (Lower-right: Overview of the investigated tissue) (**b**)

The disseminated left cerebellar lesion showed enlargement (Fig. 2a) before tirabrutinib administration, and it disappeared on MRI one month after initiating daily administration of 480 mg tirabrutinib (Fig 2b). A follow-up blood test revealed grade 3 lymphocytopenia as an adverse event. However, the patient was unfortunately re-admitted to the hospital because of a high fever (≥ 38°C) and persistent grade 3 lymphocytopenia. A computed tomography (CT) scan of the chest revealed interstitial pneumonitis with diffuse bilateral interstitial infiltrations. We initiated antibiotic treatment with tazobactam and piperacillin hydrate, followed by co-trimoxazole administration with suspicion of colocalization of pneumocystis pneumonia (PCP). Despite these treatments, a follow-up CT scan confirmed exacerbation of the interstitial pneumonitis with respiratory state gradually worsening. The patient died 11 days after admission refractory to steroid pulse therapy (methylprednisolone 1000 mg/day), and an autopsy was performed 2 hours and 22 minutes later. The retrieved brain’s microscopic specimens revealed localized edema and macrophage infiltration in the bilateral frontal lobe, left temporo-occipital lobe, and left cerebellum. No viable tumor cells were identified in the entire brain, including the left cerebellum lesion, which was initially enhanced by contrast medium on MRI (Fig. 1b).

**Fig. 2:**
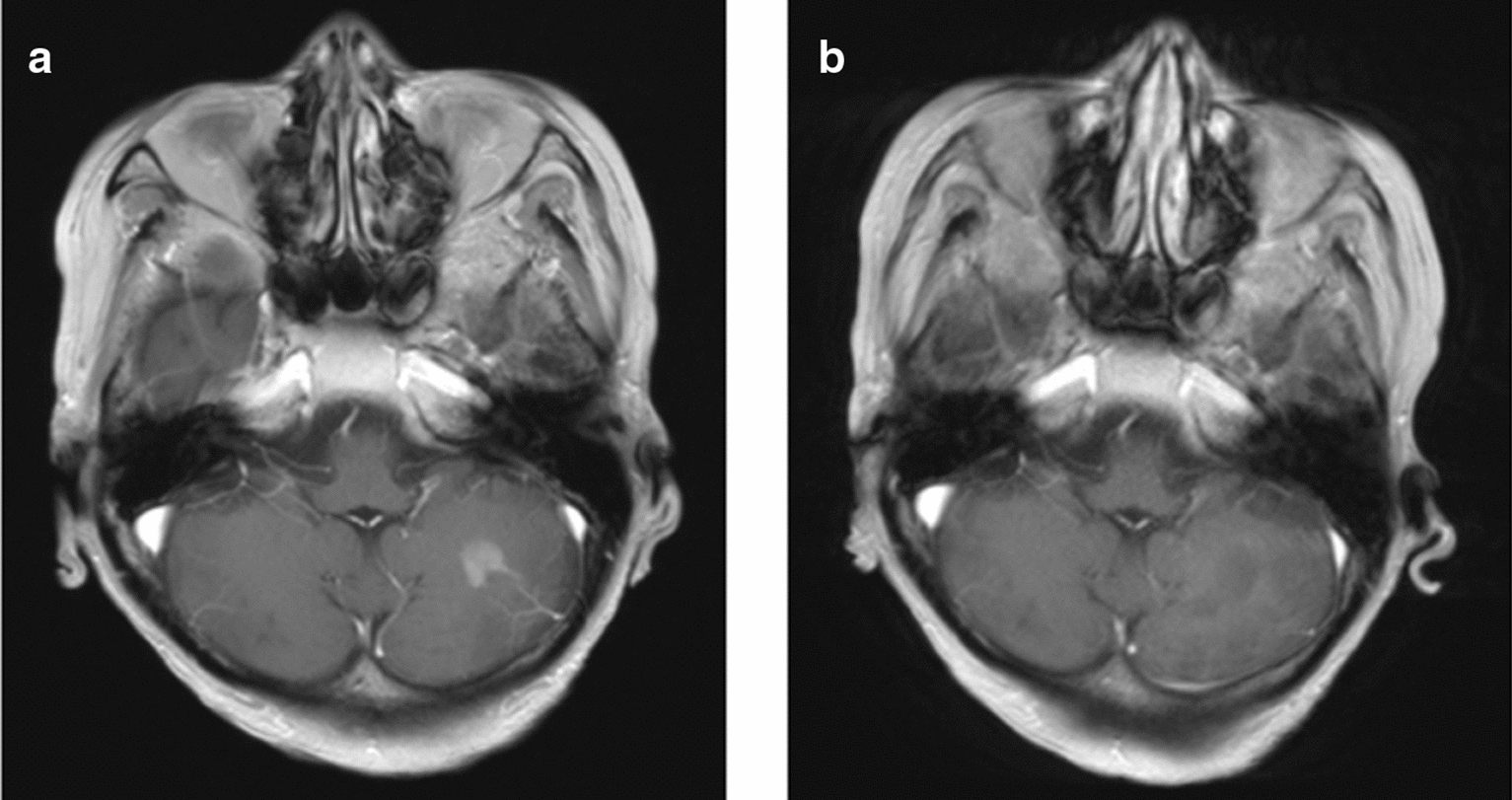
Changes of radiological appearance of the lesion on magnetic resonance imaging before and after tirabrutinib treatment are shown. The lesion was initially enhanced by a contrast agent in the left cerebellum before tirabrutinib treatment (**a)**, but the enhanced lesion disappeared one month after tirabrutinib treatment (**b)**

There is currently no standard treatment of care for relapsed/refractory PCNSL, and BTK inhibitors are one of the promising agents that may fulfill this unmet clinical need. The pivotal clinical trial for tirabrutinib approval in Japan for relapsed/refractory PCNSL revealed that the overall response rate was 64%, and the complete response rate was 34% [2]. These reported efficacies can be appreciated as high, considering that the patients enrolled in the clinical trial suffered from relapsed/refractory PCNSL. However, pathological evidence of how tirabrutinib acts against PCNSL in humans was lacking. Postmortem brain specimen revealed no viable tumor cells in the entire brain, suggesting that tirabrutinib has a direct cytotoxic effect against PCNSL. As the lesion was invisible on MRI at the time of autopsy, contrast enhancement seems to be a valuable image surrogate for monitoring lesion activity during tirabrutinib treatment. However, our observation does not provide any information on the mechanism of PCNSL becoming resistant to tirabrutinib. Studies on other types of tyrosine kinase inhibitors, such as epidermal growth factor receptor (EGFR), suggest that there could be several possible causes for tyrosine kinase inhibitor resistance, such as downregulation [3] and additional mutation within the target gene [4, 5]. Thus, it is necessary to study tirabrutinib resistant lesions in detail to search for a treatment strategy against this condition.

## Data Availability

The authors did not use any datasets, databases, or special software in writing this manuscript.

## Abbreviations

PCNSL: Primary central nervous system lymphoma
BTK: Bruton’s tyrosine kinase
